# Serum microRNA-30c levels are correlated with disease progression in Xinjiang Uygur patients with chronic hepatitis B

**DOI:** 10.1590/1414-431X20176050

**Published:** 2017-05-04

**Authors:** J. Zhang, J. Ma, H. Wang, L. Guo, J. Li

**Affiliations:** Department of Emergency, Beijing YouAn Hospital, Capital Medical University, Beijing, China

**Keywords:** Chronic hepatitis B, Disease progression, microRNA-30c, Hepatitis B virus replication, Cell proliferation

## Abstract

We aimed to investigate the potential role and mechanism of microRNA-30c (miR-30c) in the pathological development of chronic hepatitis B (CHB). The serum levels of miR-30c in hepatitis B virus (HBV) carrier Xinjiang Uygur patients with inactive, low-replicative, high-replicative and HBe antigen-positive CHB were investigated. HepG2 cells were co-transfected with pHBV1.3 and miR-30c mimic or inhibitor or scramble RNA. The effects of miR-30c dysregulation on HBV replication and gene expression, cell proliferation and cell cycle were then investigated. miR-30c was down-regulated in Xinjiang Uygur patients with CHB compared to healthy controls and its expression level discriminated HBV carrier patients with inactive, low-replicative, high-replicative and HBe antigen-positive risk for disease progression. Overexpression of miR-30c significantly inhibited HBV replication and the expressions of HBV pgRNA, capsid-associated virus DNA and Hbx in hepatoma cells. Moreover, overexpression of miR-30c significantly inhibited cell proliferation and delayed G1/S phase transition in hepatoma cells. Opposite effects were obtained after suppression of miR-30c. Our results indicate that miR-30c was down-regulated in Xinjiang Uygur patients with CHB, and miR-30c levels could serve as a marker for risk stratification of HBV infection. Down-regulation of miR-30c may result in the progression of CHB via promoting HBV replication and cell proliferation.

## Introduction

Chronic hepatitis B (CHB) is a serious worldwide public health problem caused by the infection of hepatitis B virus (HBV) (1,2). Patients with CHB have been found to exhibit a high risk of developing devastating complications, such as liver cirrhosis and hepatocellular carcinoma (3,4). Currently, detecting the enzymatic activities of some markers, such as aspartate aminotransferase and alanine aminotransferase in blood, is the most commonly used method to assess liver injury. However, the sensitivity and specificity of these markers to diagnose virus-induced liver damage are insufficient (5,6). Therefore, it is still a major clinical challenge to assess the severity of HBV-induced liver damage. Exploration of effective markers is important to better monitor the progression of CHB.

MicroRNAs (miRNAs) are evolutionarily conserved, non-coding RNAs of lengths of 20 to 25 nucleotides that can modulate gene expression of specific targets, and thus participate in various physiologic and pathologic processes (7,8). Recent findings have highlighted the pivotal roles of miRNAs in HBV-related diseases, including CHB (9–11). For instance, upregulation of miR-146a has been shown to suppress T cell function in patients with CHB and consequently contributes to immune defects during chronic viral infection (12). Serum miR-122 levels has been reported to be strongly correlated with HBs antigen and can function as a risk stratification marker to discriminate HBV carrier patients with high or low risk for disease progression (13). The elevation of serum miR-210 level is also reported to play a crucial role in liver inflammation in patients with CHB (13). Recently, miR-30c has been identified to be down-regulated in CHB patients compared to healthy donors by microarray analysis (14). However, the roles of miR-30c in regulating HBV replication and cell proliferation in the progression of CHB have not been investigated.

In this study, we investigated the serum levels of miR-30c in patients with different stages of chronic hepatitis B. Furthermore, we examined whether miR-30c dysregulation influenced HBV replication and gene expression, cell proliferation and cell cycle in hepatoma cells. Our study aimed to investigate the potential role and mechanism of miR-30c in the pathological development of CHB. Our findings will provide new insight for the diagnosis of CHB.

## Material and Methods

### Patients and samples

This study was approved by the Ethics Committee of the Beijing YouAn Hospital and was performed according to the 1964 Declaration of Helsinki.

Between April 2015 and July 2016, a total of 48 treatment-naive Xinjiang Uygur patients (D genotype 91%, B genotype 3%, and C genotype 6%) with CHB were enrolled in this study. The patients with detectable serum HBs antigen and serum HBV DNA for more than 6 months were included. Studies that are excluded fit the following criteria: within the latest 5 years, studies associated with the following research content 1) patients with organ transplantation, 2) patients with co-infections with immunodeficiency virus (HIV)/hepatitis C virus (HCV), immunosuppression, and other malignant comorbidities. Patient characteristics are summarized in Table 1. According to the HBV levels and HBe antigen status of patients and the European Association for the Study of the Liver guidelines, patients with CHB were classified into 3 groups: inactive carriers, HBe antigen-negative hepatitis (low- and high-replicative hepatitis) and HBe antigen-positive hepatitis. Low-replicative hepatitis was characterized by low viral load (HBV DNA <2000 IU/mL) and high-replicative hepatitis was characterized by high viral load (HBV DNA >2000 IU/mL). Moreover, 18 healthy subjects were included as control.

**Table 1:**
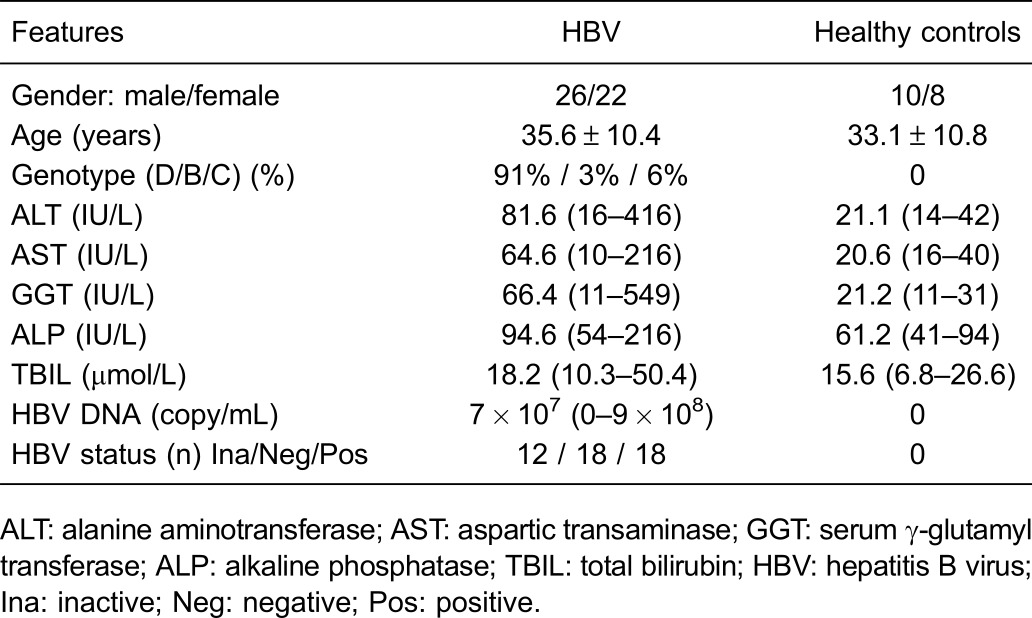
Clinical characteristics of participants.

Blood samples were collected from each individual at the time of presentation at the outpatient department and then centrifuged at 1500 *g* for 10 min at 4°C. The sera were aliquoted and then stored at -80°C until use.

### Cells culture and transfection

Human hepatoma cell line HepG2 (ATCC, USA) was maintained in Dulbecco's modified Eagle's medium (DMEM, Hi-Media Laboratories Pvt. Ltd., India), fixed with 10% fetal bovine serum (Invitrogen, USA), and treaded with penicillin-streptomycin antibiotics (Invitrogen) in a 37°C-incubator with 5% CO_2_.

After washing twice with Opti-MEM (Invitrogen), HepG2 cells were then co-transfected with pHBV1.3 and miR-30c mimic or inhibitor or scramble using Lipofectamine 2000 (Invitrogen) following the manufacturer’s recommended protocol. Each treatment was conducted in triplicate at least.

### RNA extraction and quantitative real-time PCR

Total RNA was extracted from different transfected cells using RNA Iso-plus reagent (Takara Bio, China) and then reverse transcribed into cDNA using PrimeScript RT Reagent Kit (Invitrogen) following the manufacturer's protocol. The primers in this study were designed using Primer 5.0 (Primer-E, Ltd., United Kingdom) as follows: miR-30c RT: 5′-GTCGTATCCAGTGCAGGGTCCGAGGTATTCGCACTGGATACGACGCTGAG-3′; U6 RT 5′-GTCGTATCCAGTGCAGGGTCCGAGGTATTCGCACTGGATACGACTGGAAC-3′. With a standard protocol provided by the manufacturer, qRT-PCR was performed in the ABI PRISM 7300 Fast Real-Time PCR System (Ambion, USA) using the SYBR ExScript qRT-PCR Kit (Takara, China), under the following conditions: 95°C for 5 min, 95°C for 10 s, and 40 cycles at 60°C for 30 s. Each reaction was performed in triplicate. The expression levels of miR-30c were normalized to U6. The results of RT-PCR are reported as 2^-ΔΔCt^.

### Western blot analysis

Cells were lysed using mammalian protein extraction reagent and HALT protease inhibitor cocktail (Thermo Scientific, USA). Then, the supernatant was collected and its protein content was measured using bicinchoninic acid reagent (Thermo Scientific). The equal amount of protein samples were separated using 10% SDS-PAGE and then transferred to polyvinylidene fluoride membranes (Millipore). Membranes were blocked with Tris-buffered saline (TBS) containing 5% non-fat dry milk for 1 h, and then probed using antibodies against HBx (ab203540, Abcam, UK) overnight at 4°C. The membranes were then probed with horseradish-peroxidase conjugated secondary antibodies (Santa Cruz, USA) at room temperature for 1 h, and the blots were visualized using an enhanced chemiluminescence kit (Amersham, Germany). Phosphoglyceraldehyde dehydrogenase (GAPDH) was used as an internal control (Santa Cruz).

### HBV replication and gene expression analysis

HepG2 cells were grown in 6-well plates and cell culture medium was collected at 48 h post-transfection. The amount of HBsAg and HBeAg in the supernatant was determined using ELISA kits (Shanghai KeHua Biotech, China) according to the manufacturer's protocol. In addition, at 72 h post-transfection, the expression levels of HBV pgRNA and HBV DNA were determined by quantitative real-time PCR (qRT-PCR) analysis. Briefly, cells were lysed in lysis buffer (50 mM Tris, pH 7.5, 100 mM NaCl, 1 mM EDTA and 0.5% Nonidet P-40) at 4°C for 1 h. Then, cells were incubated with MgCl_2_ and DNase I (10 mg/mL, Takara) at 37°C for 2 h to remove DNA that was not protected by the HBV core protein. Viral cores were then precipitated by centrifugation at 8,000 *g* for 5 min at 37°C after adding 0.5 M EDTA and 35% polyethylene glycol. The pellet was then resuspended in buffer A (10 mM Tris, 1 mM EDTA, 1 mM EDTA, 100 mM NaCl, 1% SDS, and 2.5 mg/mL proteinase K) for 16 h. Subsequently, capsid-associated viral DNA, which was released from the lysed cores, isolated using phenol and chloroform, precipitated with isopropyl alcohol and finally quantified with qRT-PCR analysis. The primers for HBV DNA detection were as follows: forward, 5′-AGAAACAACACATAGCGCCTCAT-3′, reverse, 5′-TGCCCCATGCTGTAGATCTTG-3′, and the HBV probe 5′-TGTGGGTCACCATATTCTTGGG-3′. All samples were analyzed in triplicate and the relative HBV DNA levels were determined after converting the viral copy numbers to fold-changes.

### Cell proliferation analysis by MTT and colony formation assays

For MTT assay, cells (3000 cells/well) were plated onto 96-well plates after transfection and continued to culture for 1-5 days. At 0, 24, 48, and 72 h after transfection, the MTT reagent (AMRESCO, USA) was added to incubate cells for 4 h at 37°C. The supernatants were then removed, and DMSO (150 μL/well) was added to dissolve the formazan crystals. The absorbance value of each well at 450 nm was measured using a multilabel plate reader (PerkinElmer, USA).

For colony formation assay, cells at a density of 100 cells/dish were placed on the 60 mm culture dishes after 48 h of transfection and maintained in complete medium for 2 weeks. Colonies were then fixed with methanol, stained with 0.1% crystal violet and counted under a microscope (IX83, Olympus, Japan). Cell number in each colony was at least 30.

### Cell cycle analysis

For cell cycle analysis, HepG2 cells transfected with miR-30c mimic, inhibitor and scramble control were cultured for 48 h. Then cells were fixed in 70% ethanol at 4°C. After washing, fixed cells were incubated in PBS mixed with 20 μg/mL of propidium iodide, 200 μg/mL of RNasemA and 0.1% Triton X-100 (BD Biosciences, USA) at 37°C for 20 min. Finally, the stained cells were analyzed using flow cytometry with the BD FACSCalibur™ system (BD Biosciences) for cell cycle distribution.

### Statistical analysis

All experiments were carried out in triplicate. Data from multiple experiments are reported as means±SD. The difference between the two groups was then compared by Student's two-tailed unpaired *t*-test, and among three or more groups by one-way analysis of variance with Bonferroni's multiple comparison test. A value of P<0.05 indicated statistically significant differences.

## Results

### Serum levels of miR-30c in patients with different stages of CHB

**Figure 1:**
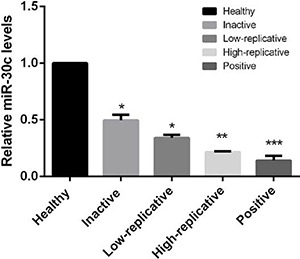
Serum levels of miR-30c in healthy controls and hepatitis B virus (HBV) carrier patients with inactive, low-replicative, high-replicative and HBe antigen-positive chronic hepatitis B. Data are reported as means±SD. *P<0.05, **P<0.01, ***P<0.001 compared to healthy controls (ANOVA).

As shown in Figure 1, the expression levels of miR-30c in patients with CHB were significantly lower than in healthy controls (P<0.05). In addition, the expression levels of miR-30c were gradually decreased in healthy controls, inactive carriers, low-replicative hepatitis, high-replicative hepatitis and HBe antigen-positive hepatitis, with significant differences (P<0.05), indicating that miR-30c might be a potential marker for the diagnosis or risk stratification of HBV infection.

### Aberrant expression of miR-30c regulated HBV replication and gene expression in hepatoma cells

**Figure 2:**
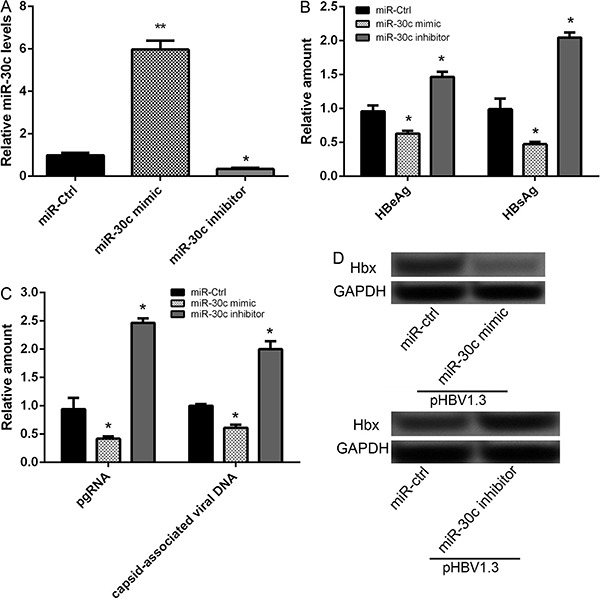
Overexpression of miR-30c inhibited HBV replication and gene expression in hepatoma cells. *A*, miR-30c expression in hepatoma cells after transfection with miR-30c mimic, inhibitor or scramble control. *B*, Amount of HBeAg and HBsAg in hepatoma cells after co-transfection with pHBV1.3 and 50 nM of miR-30c mimic, inhibitor or scramble control for 48 h. *C*, Expression levels of HBV pgRNA and capsid-associated virus DNA in hepatoma cells after co-transfection with pHBV1.3 and miR-30c mimic, inhibitor or scramble control for 72 h. *D*, Expression levels of Hbx protein in hepatoma cells after co-transfection with pHBV1.3 and miR-30c mimic, inhibitor or scramble control for 48 h. Data are reported as means±SD. *P<0.05, **P<0.01 compared to scramble controls (ANOVA).

miR-30c expression was significantly increased in hepatoma cells transfected with miR-30c mimic compared to miR-30c scramble control group, while markedly decreased in hepatoma cells transfected with miR-30c inhibitor (P<0.05), indicating that miR-30c was successfully overexpressed and suppressed in hepatoma cells (Figure 2A). After hepatoma cells were co-transfected with pHBV1.3 and 50 nM of miR-30c mimic for 48 h, the results of ELISA showed that the amount of HBeAg and HBsAg was significantly decreased compared to that in cells co-transfected with pHBV1.3 and miR-30c scramble control (P<0.05, Figure 2B). Furthermore, the results of qRT-PCR analysis showed that the expression levels of HBV pgRNA and capsid-associated virus DNA was also significantly decreased after co-transfection with pHBV1.3 and miR-30c mimic for 72 h (P<0.05, Figure 2C). The results of western blot analysis showed that the expression levels of Hbx protein were also significantly decreased after co-transfection with pHBV1.3 and miR-30c mimic for 48 h (P<0.05, Figure 2D). After cells were co-transfected with pHBV1.3 and miR-30c inhibitor, opposite effects were obtained in the amount of HBeAg and HBsAg, in mRNA expression levels of HBV pgRNA and capsid-associated virus DNA, and in the protein expression levels of Hbx (P<0.05, Figure 2B-D).

### Overexpression of miR-30c inhibited cell proliferation in hepatoma cells

**Figure 3:**
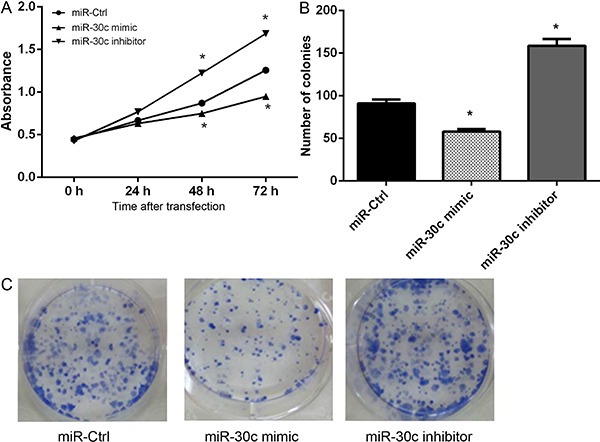
Overexpression of miR-30c inhibits cell proliferation in hepatoma cells. *A*, MTT assay shows the cell viability of hepatoma cells after transfection with miR-30c mimic, inhibitor or scramble control. *B*-*C*, Colony formation assay shows the number of colonies after hepatoma cells were transfected with miR-30c mimic, inhibitor or scramble control.

The results of MTT assay showed that, compared to miR-30c scramble transfected cells, the viability of miR-30c mimic-transfected cells significantly decreased after 48 and 72 h of transfection, while the viability of miR-30c inhibitor-transfected cells markedly increased (P<0.05, Figure 3A). In addition, the results of colony formation assay were consistent with the MTT assay results and showed that the number of colonies was significantly decreased in miR-30c mimic transfected group and increased in miR-30c inhibitor transfected group (P<0.05, Figure 3B).

### Overexpression of miR-30c induced G1/S cell cycle arrest in hepatoma cells

**Figure 4:**
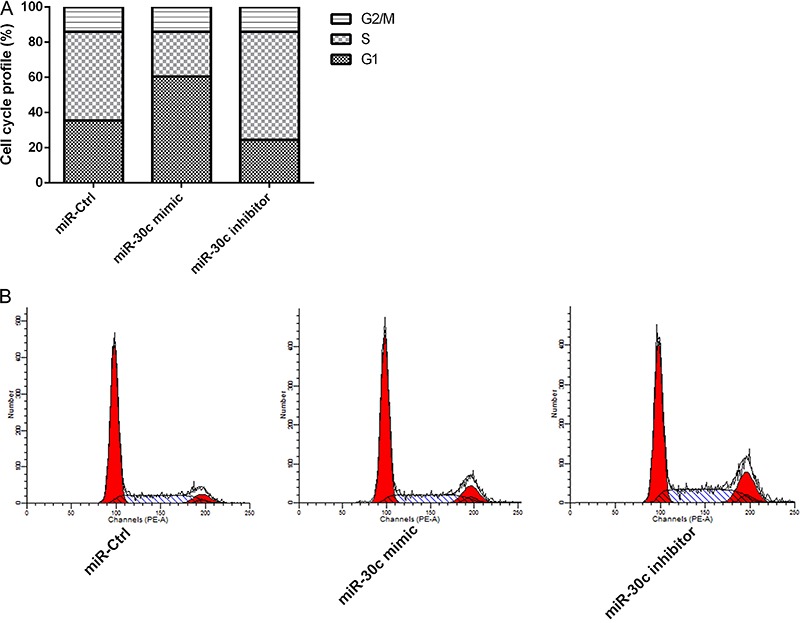
Overexpression of miR-30c induced G1/S cell cycle arrest in hepatoma cells.

The effects of miR-30c on cell cycle dysregulation were also investigated in our study. As shown in Figure 4, miR-30c mimic-transfected hepatoma cells at G1 phase were significantly increased compared to scramble transfected hepatoma cells at G1 phase, while miR-30c mimic-transfected hepatoma cells at S phase were decreased. Furthermore, hepatoma cells at G1 phase were significantly decreased after transfection with miR-30c inhibitor, while cells at S phase were markedly increased. These data indicated that overexpression of miR-30c induced G1/S cell cycle arrest in hepatoma cells.

## Discussion

This study investigated the interaction between miR-30c and HBV. The data presented here showed that miR-30c expression was significantly down-regulated in patients with CHB and its expression level was negatively correlated with the extent of HBV replication. In addition, *in vitro* cell experiments showed that overexpression of miR-30c significantly inhibited HBV replication and the expressions of HBV pgRNA, capsid-associated virus DNA and Hbx in hepatoma cells, as well as suppressed cell proliferation and induced G1/S cell cycle arrest in hepatoma cells.

Increasing evidence has shown that miRNAs play vital roles in the diagnosis, pathogenesis and therapeutic aspects of viral infection (15). A recent study has also shown that cellular and viral miRNAs can function as a new class of regulators in viral pathogenesis (16). Cellular miRNAs have the potential to promote viral replication in host cells (17). Furthermore, blood-derived microRNAs are considered to be new potential markers for the assessment of disease severity (18). Serum levels of microRNAs, such as miR-572, -575 and -744 can specifically predict liver injury in patients with CHB (14). Yu et al. (19) demonstrated that serum miR-181b was correlated with HBV replication as well as disease progression in patients with CHB. Huang et al. (20) reported that serum miR-29 was correlated with liver fibrotic stages and necroinflammation grades, and thus may be a biomarker for predicting disease progression in CHB patients. There is also evidence that down-regulation of miR-30c results in the activation of hepatitis C virus core protein-induced epithelial-mesenchymal transition, and thus may serve as a marker for poor prognosis of hepatocellular carcinoma (21). In our study, we determined that miR-30c expression was significantly down-regulated in patients with CHB compared to healthy controls. Also, serum miR-30c levels discriminated HBV carrier patients with inactive, low-replicative, high-replicative and HBe antigen-positive risk for disease progression. These data suggest that miR-30c may be negatively correlated with disease progression and could be a potential marker for risk stratification of HBV infection. Besides, miR-30c can attenuate HBV replication in hepatoma cells, which provides evidence for the role of miR-30c in HBV replication in CHB.

Furthermore, the Hbx protein of HBV has been shown to modulate cell cycle in cultured primary human hepatocytes (22). It is also reported that Hbx can activate the ATM-Chk2 pathway in HBV pathogenesis, inducing cell-cycle delay (23). Shukla et al. (24) also demonstrated that the Hbx oncoprotein of HBV could deregulate cell cycle by regulating the cellular deubiquitinase USP37. In addition, cell proliferation allows orderly progression through the cell cycle (25,26). Hbx protein has shown to regulate the proliferation of hepatocellular carcinoma cells (27). Furthermore, miR-30c was down-regulated in non-small cell lung cancer cells and can inhibit cancer cell proliferation (28). In our study, overexpression of miR-30c resulted in a significantly decreased Hbx protein level in hepatoma cells. Moreover, overexpression of miR-30c significantly inhibited cell proliferation and delayed G1/S phase transition in hepatoma cells. Although the roles of miR-30c in regulating cell proliferation in HBV pathogenesis are unclear, based on our results, it can be speculated that the decreased expression of Hbx caused by miR-30c overexpression may delay the cell cycle, inhibiting cell proliferation.

In conclusion, our results indicate that miR-30c is down-regulated in Xinjiang Uygur patients with CHB and miR-30c levels could serve as a marker for risk stratification of HBV infection. Down-regulation of miR-30c may result in the progression of CHB via promoting HBV replication and cell proliferation.
